# Nine-year risk stratification and prediction of *Helicobacter pylori* infection using Group-Based Trajectory Modeling and machine learning in 35,206 adults

**DOI:** 10.3389/fpubh.2025.1688708

**Published:** 2025-11-13

**Authors:** Heping Zhao, Sainan Liu, Manzhen Wei, Yuhan Wang, Tong Xiao, Tian Yao

**Affiliations:** 1 Department of Gastroenterology, The First Hospital of Shanxi Medical University, Taiyuan, Shanxi, China; 2 Academy of Medical Sciences, Shanxi Medical University, Taiyuan, Shanxi, China; 3 School of Public Health, Shanxi Medical University, Taiyuan, Shanxi, China; 4 First Clinical Medical College, Shanxi Medical University, Taiyuan, China

**Keywords:** *Helicobacter pylori*, machine learning, risk prediction, group-based trajectory modeling, SHapley Additive exPlanations

## Abstract

**Background:**

*Helicobacter pylori* (*H. pylori*) infection remains prevalent in regions such as Shanxi, China, contributing to gastrointestinal morbidity. Accurately identifying high-risk individuals is essential for effective screening and early intervention.

**Methods:**

We conducted a retrospective longitudinal cohort study of 35,206 adults who underwent repeated annual health checkups with *H. pylori* testing at a single center from 2016 to 2024. Group-Based Trajectory Modeling (GBTM) identified risk subgroups. Multivariable logistic regression identified predictors of high-risk trajectories; alcohol consumption was assessed as an effect modifier. Five machine learning models—including Light Gradient Boosting Machine (LightGBM), Extreme Gradient Boosting, Logistic regression, etc.—were trained using a 7:3 split. Temporal validation (2016–2020 training/2021–2024 validation) assessed generalizability. SHapley Additive exPlanations (SHAP) improved interpretability. A prediction tool was deployed via R Shiny.

**Results:**

GBTM identified high-risk (14.63%) and low-risk (85.37%) groups. Protective factors included women (OR = 0.042, 95% CI: 0.039–0.046) and unmarried status (OR = 0.092, 95% CI: 0.085–0.099); risk factors included obesity (OR = 1.138, 95% CI: 1.070–1.210), blue-collar workers (OR = 1.557, 95% CI: 1.454–1.666), and alcohol consumption (OR = 1.277, 95% CI: 1.165–1.401). Alcohol consumption interacted with all significant factors in subgroup analysis (all *p* < 0.001), with the strongest interaction observed for being married (OR = 8.622, 95% CI: 7.872–9.437). LightGBM achieved AUCs of 0.851 (training), 0.843 (validation), 0.863 (temporal training), and 0.831 (temporal validation). SHAP ranked marital status and sex as top predictors. The tool is available at: https://prediction-model-for-hp.shinyapps.io/hp_shinyapp-/.

**Conclusion:**

We developed an online, interpretable risk prediction tool with validated accuracy to support precision screening of *H. pylori* infection.

## Introduction

*Helicobacter pylori* (*H. pylori*), classified as a Group 1 carcinogen by the World Health Organization (1), is a major etiological agent of gastritis, peptic ulcer disease, and gastric cancer (GC) (2). It poses a substantial global public health burden (3). Although infection rates have declined in certain regions due to improved hygiene and widespread eradication efforts, recent epidemiological data estimate that approximately 40% of adults worldwide—and 40.7% in China—remain infected (4). This underscores the ongoing need for effective detection and management strategies (5). Given the robust evidence that *H. pylori* eradication in asymptomatic individuals significantly reduces the incidence of GC (6, 7), the early identification of high-risk populations remains a critical public health priority.

Although risk prediction models for *H. pylori* infection have been previously proposed, most are built on traditional regression methods and fail to account for heterogeneity in individual risk trajectories (8). These models often assume that all individuals follow a similar risk pattern, overlooking potential subgroups within the population who may exhibit distinct risk dynamics over time (9). In parallel, although machine learning (ML) approaches offer improved predictive performance (10), their clinical uptake remains limited by concerns around interpretability (11, 12). Some early ML studies, such as Tran et al. (10), applied machine learning to *H. pylori* risk prediction without explicit interpretability frameworks. Without transparent explanation frameworks, such as SHapley Additive exPlanations (SHAP), ML models may be perceived as “black boxes,” reducing trust and applicability in healthcare contexts (13, 14).

In response to these challenges, we developed an interpretable ML–based approach to predict *H. pylori* infection risk. This approach integrates Group-Based Trajectory Modeling (GBTM) to identify latent risk subgroups, followed by a comparative evaluation of five ML algorithms optimized for predictive performance. To enhance model interpretability, SHAP were applied to quantify the relative contribution of each predictor. Finally, we implemented our findings in a web-based, interactive prediction tool using R Shiny, aiming to facilitate real-time clinical use and support more targeted screening strategies.

## Methods

### Study population

This retrospective longitudinal cohort study included adults aged ≥18 years who underwent annual routine health examinations at Shanxi Medical University First Hospital between January 2016 and June 2024 and had complete examination records for each year, including at least one *H. pylori* test per year. Each participant underwent repeated annual assessments, including *H. pylori* testing, physical examination, and structured questionnaires. Demographic and clinical data were collected at each visit via structured medical records and institutional health checkup databases.

Sex was categorized as men or women. Age groups (<50, 50–69, and ≥70 years) were defined based on the cohort’s median and mean age (48 years). Marital status was dichotomized into married versus unmarried, with the unmarried group including single, divorced, and widowed individuals (15). Body mass index (BMI) values were categorized as <24.0 and ≥24.0 kg/m^2^ based on Chinese classification standards (<18.5: underweight; 18.5–23.9: normal; 24.0–27.9: overweight; ≥28.0: obese) to enhance model interpretability and ensure statistical stability in multivariable analysis (16). Occupational classification followed the *Occupational Classification Dictionary of the People’s Republic of China* (17). Blue-collar workers included (1) workers engaged in agriculture, forestry, animal husbandry, fishery, and water conservancy production; and (2) operators and related personnel in production and transportation equipment. White-collar workers included (1) government officials, leaders of party and mass organizations, and managers in enterprises and public institutions; and (2) professional and technical personnel. Hypertension was categorized as yes or no based on systolic blood pressure ≥140 mm Hg or diastolic blood pressure ≥90 mm Hg (18). Smoking status was categorized as yes or no, with current smokers were defined as individuals who used tobacco in the past 30 days (19). Alcohol consumption was similarly categorized as yes or no, with current drinkers were defined as those who consumed alcohol at least once weekly in the past year (20). This study was approved by the Ethics Committee of Shanxi Medical University First Hospital (approval number KYLL-2024-226).

### *H. pylori* testing

During the study period, *H. pylori* infection was detected using the ^13^C-urea breath test (^13^C-UBT) (Shenzhen Headway Company) (21). Participants were instructed to fast for at least 6 h prior to testing and to rinse their mouths with water before ingesting the ^13^C-labeled urea solution. Breath samples were collected 30 min post-ingestion and analyzed via isotope ratio mass spectrometry. According to the manufacturer’s guidelines, a delta over baseline (DOB) value of ≥4.0‰ was considered indicative of active *H. pylori* infection.

### Statistical analysis

GBTM was used to classify participants into distinct *H. pylori* infection risk trajectories based on annual health checkup data from 2016 to 2024. Censored normal models with quadratic polynomial terms were applied. Models with different numbers of groups (ranging from 1 to 4) were compared using the Bayesian Information Criterion (BIC), Akaike Information Criterion (AIC), entropy, and average posterior probabilities (AvePP). The optimal number of trajectories was selected based on the lowest BIC, high entropy (≥0.80), and average posterior probabilities ≥0.70, consistent with established recommendations for GBTM.

Participants in the low-risk trajectory group (class = 0) were defined as the negative control, and those in the high-risk trajectory group (class = 1) as the positive control, based on GBTM analysis of longitudinal *H. pylori* infection status. For all ML models and subsequent risk factor analyses, these trajectory groups were used as the outcome variable, with the high-risk group serving as the positive class and the low-risk group as the negative class.

Between-group differences were assessed using the chi-square test, and variables with *p*-values <0.05 were included in multivariable logistic regression to identify independent risk factors, with results reported as odds ratios (ORs) and 95% confidence intervals (CIs). Alcohol consumption was treated as an exposure variable in subgroup analyses, and interaction effects were tested using multiplicative terms in the logistic regression models. To account for the imbalance between the high-risk and low-risk trajectory groups, a weighting scheme was applied in the logistic regression, assigning higher weights to participants in the high-risk trajectory to account for class imbalance and improve estimate stability. To control for potential inflation of type I error due to multiple subgroup and interaction tests, *p*-values were adjusted using the Benjamini–Hochberg false discovery rate (FDR) method. Stratum-specific ORs were displayed in forest plots.

The dataset was randomly divided into a training set (70%) and a validation set (30%) to develop five ML models: Light Gradient Boosting Machine (LightGBM), Extreme Gradient Boosting (XGBoost), Logistic Regression, Naive Bayes, and Elastic Net. These five models were selected for their complementary strengths with large epidemiologic datasets. The strengths and limitations of the five ML models are summarized in the Supplementary Table S1. To address potential class imbalance between trajectory groups, the Synthetic Minority Oversampling Technique (SMOTE) was applied to the training set only; the validation set was left unaltered to preserve the real-world class distribution.

Each model was implemented using standard R packages (caret, glmnet, xgboost, lightgbm, naivebayes), and hyperparameter tuning was performed with five-fold cross-validation within the training set. Model performance metrics included area under the receiver operating characteristic curve (AUC) with 95% CIs, sensitivity, specificity, and accuracy. Accuracy was calculated as the proportion of correctly classified cases among all participants, with the optimal cutoff determined by the Youden index. Additional evaluation metrics, including baseline plots, receiver operating characteristic (ROC) curves, calibration plots, and decision curve analysis (DCA), were used to assess discrimination, calibration, and clinical utility. To evaluate temporal robustness and generalizability, the dataset was temporally split, with data from 2016 to 2020 as the temporal training set and data from 2021 to 2024 as the temporal validation set. Calibration curves, decision curves, and ROC curves were generated for both validation phases.

SHAP values were applied to the best-performing model (LightGBM) to interpret the contribution and direction of each predictor. DCA was performed using the rmda package in R software to assess clinical net benefit. Finally, we developed an interactive, web-based prediction tool for *H. pylori* infection risk using R Shiny to support real-time clinical decision-making. To illustrate the overall workflow, a flowchart was generated (Supplementary Figure S1). Performance reporting and model evaluation adhered to the transparent reporting of a multivariable prediction model for individual prognosis or diagnosis guidelines (22), ensuring that discrimination, calibration, and clinical utility were consistently assessed across all models. All statistical analyses were conducted in R (version 4.4.2), and a two-sided *p*-value <0.05 was considered statistically significant.

## Results

### Baseline characteristics

A total of 35,206 individuals were included in the final analysis, including 31,512 men (89.5%) and 3,694 women (10.5%) who underwent routine health examinations and *H. pylori* testing at Shanxi Medical University First Hospital from 2016 to 2024.

GBTM was performed to identify distinct longitudinal risk trajectories of *H. pylori* infection. Models with one to four groups were compared using fit indices including the AIC, BIC, entropy, and AvePP. The two-group model showed the best balance between model fit and interpretability, exhibiting the lowest AIC (−140,350.21) and BIC (−140,220.53), acceptable entropy (0.82), and high AvePP (>0.85) for each group.

**Figure 1:**
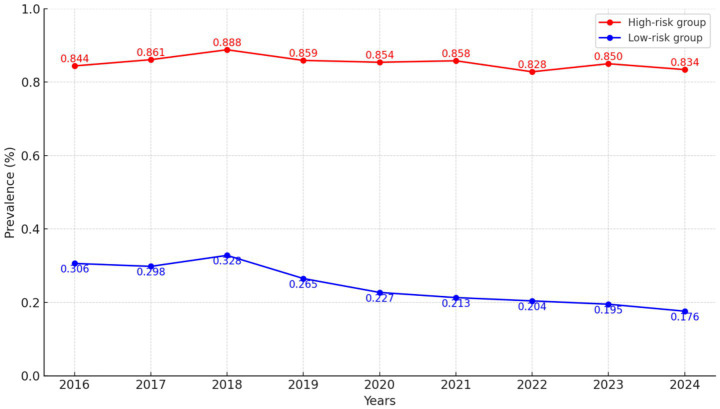
GBTM of *H. pylori* infection risk over time. Estimated probability trajectories of *H. pylori* infection from 2016 to 2024, identifying distinct risk subgroups.

Based on this model, participants were classified into a low-risk group (85.37%) and a high-risk group (14.63%) (Figure 1; Table 1). The high-risk group was characterized by a predominance of men (61.27%), younger age (≥70 years, 45.90%), higher BMI (≥24, 56.09%), married status (58.86%), white-collar occupation (71.19%), absence of hypertension (88.51%), smoking (65.27%), and alcohol consumption (75.60%). Differences between the two groups were statistically significant (all *p* < 0.001; Table 1).

**Table 1:** Baseline characteristics by *H. pylori* trajectory groups.

Factors	Low-risk group (85.37%)	High-risk group (14.63%)	*χ*^2^ value	*p*-value
Sex			5122.880	<0.001
Men	28,356 (94.35%)	3,156 (61.27%)		
Women	1,699 (5.65%)	1,995 (38.73%)		
Age (years)			5385.211	<0.001
<50	9,559 (31.81%)	2,228 (43.25%)		
50–69	17,201 (57.23%)	559 (10.85%)		
≥70	3,295 (10.96%)	2,364 (45.90%)		
Body mass index (kg/m^2^)			149.245	<0.001
<24	10,535 (35.05%)	2,262 (43.91%)		
≥24	19,520 (64.95%)	2,889 (56.09%)		
Marital status			1815.232	<0.001
Married	25,332 (84.29%)	3,032 (58.86%)		
Unmarried	4,723 (15.71%)	2,119 (41.14%)		
Occupation			341.318	<0.001
White-collar workers	17,286 (57.51%)	3,667 (71.19%)		
Blue-collar workers	12,769 (42.49%)	1,484 (28.81%)		
Hypertension			43.489	<0.001
No	25,549 (85.01%)	4,559 (88.51%)		
Yes	4,506 (14.99%)	592 (11.49%)		
Smoking			349.178	<0.001
No	6,801 (22.63%)	1,789 (34.73%)		
Yes	23,254 (77.37%)	3,362 (65.27%)		
Alcohol consumption			609.024	<0.001
No	3,508 (11.67%)	1,257 (24.40%)		
Yes	26,547 (88.33%)	3,894 (75.60%)		

### Risk factors for *H. pylori* infection and subgroup analysis

Multivariable logistic regression was conducted with trajectory group (high-risk vs. low-risk) as the dependent variable. Variables that showed significant associations in univariable analyses were included in the model. Independent risk factors associated with increased odds of *H. pylori* infection were obesity (OR = 1.138, 95% CI: 1.070–1.210), alcohol consumption (OR = 1.277, 95% CI: 1.165–1.401), and blue-collar workers (OR = 1.557, 95% CI: 1.454–1.666). A sensitivity analysis using six more detailed occupational categories yielded generally consistent results, supporting the robustness of the occupational finding (Supplementary Table S2). Conversely, women (OR = 0.042, 95% CI: 0.039–0.046) and unmarried status (OR = 0.092, 95% CI: 0.085–0.099) were associated with a lower risk of infection (Table 2). Weighted analyses were also performed to account for potential differences in trajectory group sizes, and results were consistent with the unweighted analysis (Supplementary Table S3).

**Table 2:** Multivariable logistic regression analysis of factors associated with high-risk *H. pylori* trajectory.

Factors	*β* coefficient	*p-value*	OR	95% CI
Sex (ref: men)	−3.164	<0.001	0.042	0.039–0.046
Body mass index (ref: <24 kg/m^2^)	0.129	<0.001	1.138	1.070–1.210
Marital status (ref: married)	−2.389	<0.001	0.092	0.085–0.099
Occupation (ref: White-collar workers)	0.442	<0.001	1.557	1.454–1.666
Alcohol consumption (ref: no)	0.245	<0.001	1.277	1.165–1.401

CI, confidence intervals; OR, odds ratio.

Subgroup analyses were performed stratified by alcohol consumption, treated as the exposure variable. Interaction terms between alcohol and other significant factors were tested in multivariable logistic regression models, with both subgroup-specific and interaction *p*-values adjusted for multiple testing using the Benjamini–Hochberg FDR method. Forest plots visualized stratum-specific ORs (Supplementary Figure S2). After adjustment, significant effect modification by alcohol consumption was observed in the associations between all other significant factors and *H. pylori* infection (all adjusted *p* for interaction <0.001). The strongest modification was observed for marital status: the effect of being married on infection risk was substantially amplified among drinkers (OR = 8.622, 95% CI: 7.872–9.437, adjusted *p* < 0.001).

### Model development and evaluation

The dataset was randomly divided into a training set (70%) and a validation set (30%). Baseline plots, ROC curves, calibration plots, and DCA were generated to evaluate model performance. The baseline plot showed good agreement with the results from multivariable logistic regression. All three evaluation curves (ROC, calibration, and DCA) demonstrated good performance in both the training and validation sets (Supplementary Figures S3, S4).

**Figure 2:**
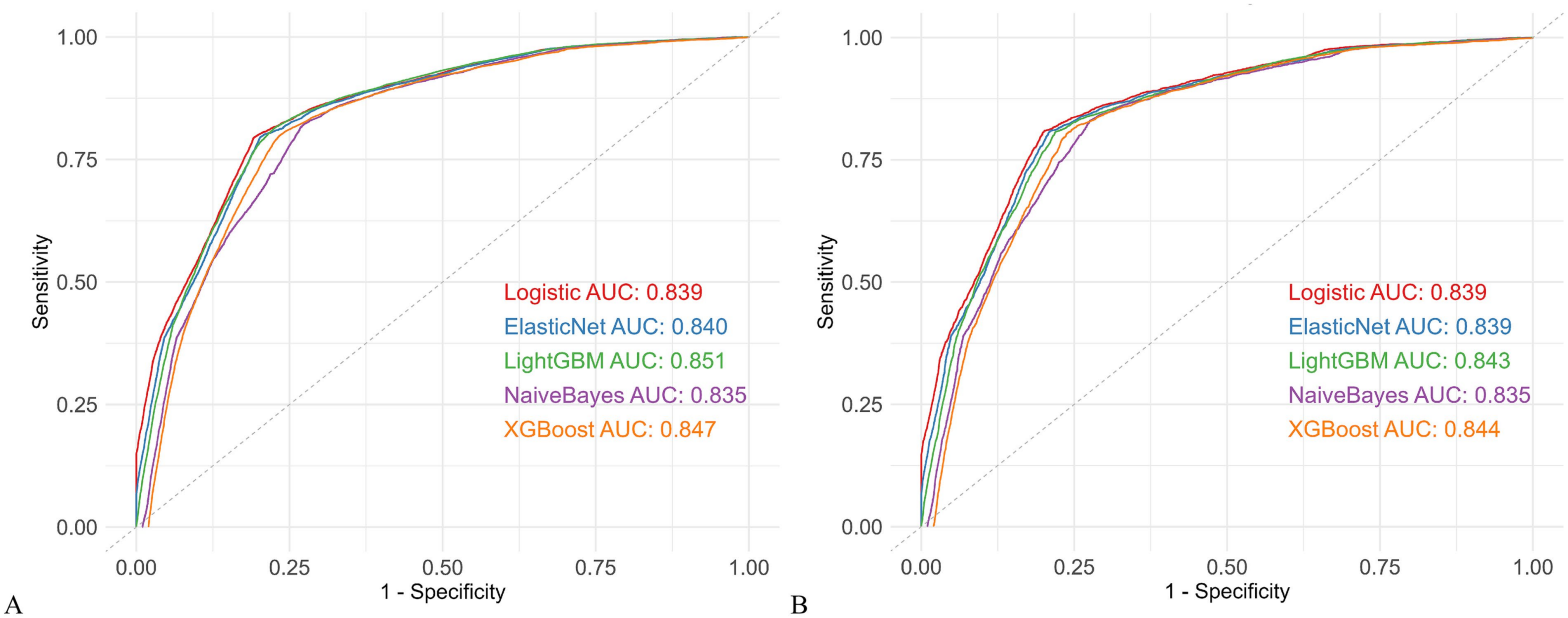
Comparison of five machine learning models. **(A)** ROC curves in the training cohort. **(B)** ROC curves in the validation cohort. AUC, area under the receiver operating characteristic curve; ROC, receiver operating characteristic; LightGBM, Light Gradient Boosting Machine; XGBoost, Extreme Gradient Boosting.

Five ML models were developed: LightGBM, XGBoost, Logistic Regression, Naive Bayes, and Elastic Net. The performance of each model in the overall training and validation sets is summarized in Supplementary Table S4, with ROC curves presented in Figure 2. LightGBM achieved the best performance, with AUCs of 0.851 (95% CI: 0.848–0.853) in the training set and 0.843 (95% CI: 0.837–0.850) in the validation set. Accuracy was 0.805/0.791, with well-balanced sensitivity (0.813/0.807) and specificity (0.775/0.781), indicating strong discriminative ability and robustness. The other models also performed well, showing stable discrimination and overall performance: XGBoost (AUC 0.847/0.844; Accuracy 0.818/0.805), Elastic Net (AUC 0.840/0.839; Accuracy 0.802/0.783), Logistic Regression (AUC 0.839/0.839; Accuracy 0.799/0.783), and Naive Bayes (AUC 0.835/0.835; Accuracy 0.826/0.769). Notably, Naive Bayes had the highest sensitivity (0.822/0.831) but slightly lower overall accuracy.

To further assess model robustness and generalizability, the dataset was temporally split, with data from 2016 to 2020 used as the temporal training set and 2021–2024 as the temporal validation set. Models were retrained, and their performance was evaluated using ROC curves, calibration plots, and DCA (Supplementary Figures S5, S6; Supplementary Table S5). LightGBM maintained the highest stability, with AUCs of 0.863 (95% CI: 0.859–0.866) in the temporal training set and 0.831 (95% CI: 0.825–0.837) in the temporal validation set, sensitivity (0.819/0.801), specificity (0.788/0.766), and accuracy (0.835/0.775). XGBoost, Elastic Net, and Logistic Regression remained stable over time (XGBoost: AUC 0.859/0.831; Accuracy 0.856/0.772; Elastic Net: AUC 0.852/0.830; Accuracy 0.795/0.790; Logistic Regression: AUC 0.852/0.830; Accuracy 0.794/0.789). Naive Bayes showed lower temporal accuracy (AUC 0.851/0.815; Accuracy 0.766/0.764).

Overall, LightGBM consistently demonstrated the best discrimination, calibration, and temporal stability among all models.

### Model interpretability

**Figure 3:**
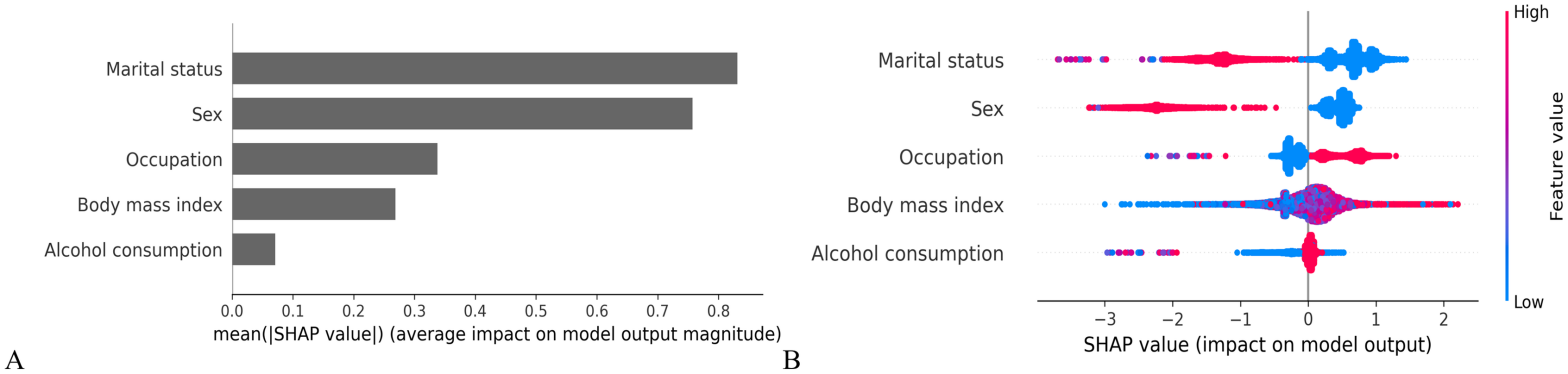
SHAP-based interpretation of model features. **(A)** Mean SHAP value bar plot (feature importance ranking). **(B)** SHAP summary plot (feature effects on model output).

To enhance clinical interpretability, SHAP values were used to quantify the contribution and directional influence of each predictor in the LightGBM model. As shown in Figure 3A, the top five predictors of *H. pylori* infection risk were marital status, sex, occupation, BMI, and alcohol consumption, ranked by overall feature importance.

Figure 3B presents the individual-level associations between each predictor and infection risk. Factors positively associated with increased risk included blue-collar workers, higher BMI and alcohol consumption. In contrast, being women and unmarried were associated with a lower predicted probability of infection, suggesting potential protective effects.

### Model deployment

Given its superior predictive performance across all datasets, the LightGBM model was selected as the core algorithm for deployment in an interactive, web-based risk prediction tool. The model was fine-tuned using five-fold cross-validated grid search, yielding the following optimal risk-enhancing input profiles: “Sex”: Men; “Marital status”: Married; “Alcohol consumption”: Yes; “Occupation”: White-collar workers; and “BMI”: ≥24.

A publicly accessible web application was developed using R Shiny: https://prediction-model-for-hp.shinyapps.io/hp_shinyapp-/. Users can enter their information through drop-down menus and click the “Predict” button to receive individualized risk estimates (low, moderate, or high) based on model-derived probabilities. For individuals identified as high risk, this tool may facilitate early identification and prompt preventive interventions for *H. pylori* infection, as shown in Supplementary Figure S7.

## Discussion

Globally, *H. pylori* infection remains a major public health concern, with approximately 43% of adults infected, particularly in regions such as China (4, 23). Although previous studies have explored its risk factors using cross-sectional designs or traditional regression methods, few have applied dynamic classification approaches or interpretable ML models to track risk over time in large longitudinal cohorts (10).

In this study, we analyzed a longitudinal cohort of 35,206 individuals with repeated annual health checkups from 2016 to 2024. This design not only enabled risk identification and early intervention but also provided high-quality longitudinal data for refining predictive models and evaluating long-term public health interventions (24–27). To our knowledge, this is the first study to integrate GBTM with both logistic regression and ML methods for dynamic risk stratification, further translated into a web-based tool for individualized prediction.

Obesity emerged as an independent risk factor. Mechanistically, excess adiposity alters gut microbiota composition (28), particularly by increasing the Firmicutes-to-Bacteroidetes ratio, which may promote *H. pylori* colonization (29, 30). Obesity-related dysbiosis also impairs mucosal immune defenses and disrupts gastric barrier function (31), while chronic low-grade inflammation and insulin resistance further weaken host immunity (32, 33). These findings align with recent microbiome studies linking obesity to increased epithelial permeability, reduced antimicrobial peptide production, and a weakened gastric mucosal barrier (34). BMI thus serves not only as a marker of adiposity, but also as a clinically relevant proxy for a broader set of physiological, behavioral, and metabolic factors—including diet, physical activity, and microbiome-associated immune modulation—that together shape *H. pylori* susceptibility (34, 35).

Occupational status was another important factor. Blue-collar workers —including those in agriculture, manufacturing, and transportation—had significantly higher *H. pylori* infection risk than white-collar workers. This may result from greater exposure to suboptimal sanitation, limited access to clean water, and communal dining practices (36, 37). Socioeconomic disadvantage and lower health literacy may further exacerbate vulnerability (38). Beyond direct exposure, occupational status may also act as a proxy for broader social determinants of health—such as hygiene awareness, nutritional quality, and chronic stress—that collectively shape an individual’s susceptibility to persistent *H. pylori* colonization (39, 40).

Alcohol consumption emerged as both a direct risk factor and an effect modifier in interactions with other significant variables. Prior studies have reported a dose-dependent association: light-to-moderate intake is associated with lower infection odds, while heavy drinking impairs immune defenses and gastric barrier integrity, increasing susceptibility to colonization (41–44). This may be attributed to ethanol metabolites, such as acetaldehyde, which damage the gastric mucosa and compromise the gastric barrier (45). Additionally, alcohol suppresses immune responses, modulates gut microbiota by reducing beneficial bacteria and promoting pathogenic bacteria growth, and suppresses antimicrobial peptide production, all of which enhance *H. pylori* colonization (46–48). In our study, alcohol also demonstrated interactive effects with sex, marital status, occupational status, and BMI, suggesting potential synergism in modulating susceptibility to *H. pylori* infection.

Conversely, women and unmarried individuals were protective factors. The lower infection risk among women may be attributed to physiological differences, particularly the influence of sex hormones, which modulate immune and inflammatory responses and alter the host’s immune reaction to *H. pylori* infection (49). Additionally, women tend to be more vigilant about hygiene practices, including frequent handwashing and food preparation hygiene, which likely reduce the risk of fecal–oral transmission (50, 51). Gender roles in family settings, where women often assume household care responsibilities, may reinforce these protective practices (52). Unmarried individuals may experience reduced intrafamilial transmission, as *H. pylori* is commonly spread among spouses (53, 54). One study found that over 68% of infected couples shared identical strains, with risk increasing with marriage duration (55). Reduced shared meals, less close contact, and dispersed social networks likely explain the lower prevalence among unmarried groups (56, 57).

Among the ML models, LightGBM achieved the highest and most stable performance, with AUCs of 0.851 (training set), 0.843 (validation set), 0.863 (temporal training set), and 0.831 (temporal validation set). SHAP confirmed marital status, sex, occupation, BMI, and alcohol consumption as the top predictors, aligning with multivariable regression and enhancing model interpretability.

Prior work has demonstrated the utility of LightGBM in gastroenterology: Wang et al. (58) predicted postoperative complications in GC (AUC = 0.923, accuracy 87.3%); Fu et al. (59) used it for GC screening in 25,622 participants with high recall (94.6%) even without *H. pylori* IgG data; and Yang et al. (60) predicted esophageal cancer surgery complications with excellent discrimination (AUC = 0.956). Our study extends these findings by applying LightGBM to a significantly larger longitudinal cohort and including both temporal validation. The model not only achieved high discrimination but also offered interpretability through SHAP values and a practical, web-based tool for real-time risk stratification. While the Shiny application provides an accessible platform to estimate individual *H. pylori* infection risk (categorizing users as low, moderate, or high risk), broader validation in multi-center cohorts will be necessary before clinical integration. If validated prospectively, such a tool may assist clinicians as a pre-screening aid to help prioritize individuals for confirmatory testing.

### Strengths and limitations

This study offers several strengths. First, it employed GBTM to capture dynamic risk trajectories over time. Second, the large sample size and extended follow-up period enhance statistical power and model robustness. Third, combining interpretable ML with conventional regression balances predictive accuracy with clinical relevance. Fourth, the resulting R Shiny-based prediction tool enables accessible, real-time individualized screening in primary care and public health settings.

However, several limitations should be noted. Although variables such as BMI, occupation, and alcohol consumption may indirectly capture aspects of lifestyle and socioeconomic status due to their strong associations with factors like diet, income, and hygiene (20, 35, 39), the absence of direct measurement of these lifestyle indicators may still limit the comprehensiveness of the risk assessment. Such omissions may lead to residual confounding and potentially bias the estimated associations (61). Future studies should incorporate more detailed predictors and conduct multicenter external validation. Additionally, integration with mobile platforms or electronic health records could further support early detection and individualized risk management.

## Conclusion

This study is the first to combine GBTM with multiple ML methods to identify and validate key risk factors for *H. pylori* infection. The resulting interpretable and high-performing model was translated into a user-friendly online tool, providing a scalable solution for personalized prevention. Future work should include prospective studies to evaluate the impact of early intervention in high-risk individuals and assess model performance across diverse settings.

## Data Availability

The raw data supporting the conclusions of this article will be made available by the authors, without undue reservation.
