# Supplementary material for: Nine-year risk stratification and prediction of Helicobacter pylori infection using Group-Based Trajectory Modeling and machine learning in 35,206 adults
Source: Front Public Health. 2025 Nov 13;13:1688708. doi: 10.3389/fpubh.2025.1688708 (PMC12657407; doi:10.3389/fpubh.2025.1688708)

**SUPPLEMENT.**

**eTable 1.** Strengths and limitations of the five ML models used in this study.

**eTable 2.** Sensitivity analysis of *H. pylori* high-risk trajectory using six occupational categories.

**eTable 3.** Weighted multivariable logistic regression analysis of factors associated with high-risk *H. pylori* trajectory.

**eTable 4.** Performance of five ML models in the overall training and validation sets.

**eTable 5.** Performance of five ML models in the temporal training (2016–2020) and temporal validation (2021–2024) sets.

**eFigure 1.** Workflow of data processing, trajectory modeling, and ML model development for *H. pylori* infection prediction.

**eFigure 2.** Subgroup analysis stratified by alcohol consumption.

**eFigure 3.** Model performance in the training cohort. (A) Baseline trajectory plot; (B) ROC curve; (C) Calibration curve; (D) DCA.

**eFigure 4.** Model performance in the validation cohort. (A) ROC curve; (B) Calibration curve; (C) DCA.

**eFigure 5.** Temporal training set evaluation (2016–2020). (A) Calibration curve; (B) DCA; (C) ROC curves for five ML models.

**eFigure 6.** Temporal validation set evaluation (2021–2024). (A) Calibration curve; (B) DCA; (C) ROC curves for five ML models.

**eFigure 7.** Web-based prediction tool interface.

**Supplemental Online Content**

**eTable 1.** Strengths and limitations of the five ML models used in this study.

**eTable 2.** Sensitivity analysis of *H. pylori* high-risk trajectory using six occupational categories.

**eTable 3.** Weighted multivariable logistic regression analysis of factors associated with high-risk *H. pylori* trajectory.

**eTable 4.** Performance of five ML models in the overall training and validation sets.

**eTable 5.** Performance of five ML models in the temporal training (2016–2020) and temporal validation (2021–2024) sets.

**eFigure 1.** Workflow of data processing, trajectory modeling, and ML model development for *H. pylori* infection prediction**.**

**eFigure 2.** Subgroup analysis stratified by alcohol consumption.

**eFigure 3.** Model performance in the training cohort. (A) Baseline trajectory plot; (B) ROC curve; (C) Calibration curve; (D) DCA.

**eFigure 4.** Model performance in the validation cohort. (A) ROC curve; (B) Calibration curve; (C) DCA.

**eFigure 5.** Temporal training set evaluation (2016–2020). (A) Calibration curve; (B) DCA; (C) ROC curves for five ML models.

**eFigure 6.** Temporal validation set evaluation (2021–2024). (A) Calibration curve; (B) DCA; (C) ROC curves for five ML models.

**eFigure 7.** Web-based prediction tool interface.

This supplemental material has been provided by the authors to give readers additional information about their work.

**eTable 1. Strengths and limitations of the five ML models used in this study.**

| **Models** | **Strengths** | **Limitations** | **Reference** |
| --- | --- | --- | --- |
| LightGBM | Efficient gradient boosting; handles large datasets and categorical variables | Less interpretable; sensitive to parameter tuning | Babette A B, Zhuangyu C, Amy B D. *Methods of estimating or accounting for neighborhood associations with health using complex survey data.* Am J Epidemiol 2014; 179(10). |
| XGBoost | Strong predictive performance; tree-ensemble boosting | Less interpretable; sensitive to hyperparameter tuning | Daniel W, Justin L, Michele Jonsson F. *Propensity score estimation: neural networks, support vector machines, decision trees (CART), and meta-classifiers as alternatives to logistic regression.* J Clin Epidemiol 2010; 63(8). |
| Elastic Net | Handles multicollinearity; reduces overfitting | Limited to linear relationships; may miss nonlinear associations | Ulrich G, Helmut K, Peter S, Ralf B. *Flexible regression models are useful tools to calculate and assess threshold values in the context of minimum provider volumes.* J Clin Epidemiol 2008; 61(11). |
| Logistic Regression | Widely used baseline linear model; interpretable; suitable for binary outcomes | May overlook complex dependencies or time-varying effects | Martin W, Jan B, Petra G, Martin S. *Modeling the effect of time-dependent exposure on intensive care unit mortality.* Intensive Care Med 2009; 35(5). |
| Naive Bayes | Fast probabilistic model; ideal for large datasets | Assumes feature independence, reducing accuracy if predictors are correlated | J V T. *Advantages and disadvantages of using artificial neural networks versus logistic regression for predicting medical outcomes.* J Clin Epidemiol 1996; 49(11). |

Abbreviations: ML, machine learning; LightGBM, Light Gradient Boosting Machine; XGBoost, Extreme Gradient Boosting.

**eTable 2. Sensitivity analysis of *H. pylori* high-risk trajectory using six occupational categories.**

| **Factors** | ***β Coefficient*** | ***P-value*** | **OR** | **95% CI** |
| --- | --- | --- | --- | --- |
| Sex (Ref: Men) | -2.535 | <0.001 | 0.079 | 0.074-0.085 |
| Body mass index (Ref: <24 kg/m²) | 0.085 | 0.001 | 1.089 | 1.034-1.147 |
| Marital status (Ref: Married) | -2.927 | <0.001 | 0.054 | 0.049-0.059 |
| Occupation (Ref: Enterprise employee) |  |  |  |  |
| Teacher | -0.100 | 0.022 | 0.905 | 0.831-0.986 |
| Administrative worker | -0.048 | 0.302 | 0.953 | 0.871-1.044 |
| Healthcare professional | -1.538 | <0.001 | 0.215 | 0.194-0.237 |
| Worker | 0.498 | <0.001 | 1.645 | 1.414-1.914 |
| Farmer | 0.724 | <0.001 | 2.062 | 1.731-2.456 |
| Alcohol consumption (Ref: No) | 0.121 | 0.004 | 1.129 | 1.041-1.225 |

Abbreviations: CI: confidence intervals. OR: odds ratio.

**eTable 3. Weighted multivariable logistic regression analysis of factors associated with high-risk *H. pylori* trajectory.**

| **Factors** | ***β Coefficient*** | ***P-value*** | **OR** | **95% CI** |
| --- | --- | --- | --- | --- |
| Sex (Ref: Men) | -3.121 | <0.001 | 0.044 | 0.042-0.046 |
| Body mass index (Ref: <24 kg/m²) | 0.245 | <0.001 | 1.278 | 1.242-1.315 |
| Marital status (Ref: Married) | -2.468 | <0.001 | 0.085 | 0.082-0.088 |
| Occupation (Ref: White-collar workers) | 0.755 | <0.001 | 2.128 | 2.063-2.194 |
| Alcohol consumption (Ref: No) | 0.428 | <0.001 | 1.534 | 1.468-1.602 |

Abbreviations: CI: confidence intervals. OR: odds ratio.

**eTable 4. Performance of five ML models in the overall training and validation sets.**

| **Models** | **Dataset** | **Sensitivity** | **Specificity** | **Accuracy** | **AUC** | **95% CI** |
| --- | --- | --- | --- | --- | --- | --- |
| LightGBM | Training | 0.813 | 0.775 | 0.805 | 0.851 | 0.848 - 0.853 |
|  | Validation | 0.807 | 0.781 | 0.791 | 0.843 | 0.837 - 0.850 |
| XGBoost | Training | 0.799 | 0.786 | 0.818 | 0.847 | 0.843 - 0.852 |
|  | Validation | 0.806 | 0.780 | 0.805 | 0.844 | 0.837 - 0.850 |
| Elastic Net | Training | 0.797 | 0.787 | 0.802 | 0.840 | 0.837 - 0.843 |
|  | Validation | 0.809 | 0.779 | 0.783 | 0.839 | 0.832 - 0.846 |
| Logistic Regression | Training | 0.795 | 0.789 | 0.799 | 0.839 | 0.836 - 0.842 |
|  | Validation | 0.809 | 0.779 | 0.783 | 0.839 | 0.832 - 0.846 |
| Naive Bayes | Training | 0.822 | 0.736 | 0.826 | 0.835 | 0.833 - 0.839 |
|  | Validation | 0.831 | 0.734 | 0.769 | 0.835 | 0.828 - 0.841 |

Abbreviations: AUC, area under the receiver operating characteristic curve; CI, confidence interval; ML, machine learning; LightGBM, Light Gradient Boosting Machine; XGBoost, Extreme Gradient Boosting.

**eTable 5. Performance of five ML models in the temporal training (2016–2020) and temporal validation (2021–2024) sets.**

| **Models** | **Dataset** | **Sensitivity** | **Specificity** | **Accuracy** | **AUC** | **95% CI** |
| --- | --- | --- | --- | --- | --- | --- |
| LightGBM | Training | 0.819 | 0.788 | 0.835 | 0.863 | 0.859 - 0.866 |
|  | Validation | 0.801 | 0.766 | 0.775 | 0.831 | 0.825 - 0.837 |
| XGBoost | Training | 0.801 | 0.803 | 0.856 | 0.859 | 0.854 - 0.864 |
|  | Validation | 0.798 | 0.771 | 0.772 | 0.831 | 0.825 - 0.836 |
| Elastic Net | Training | 0.816 | 0.787 | 0.795 | 0.852 | 0.849 - 0.856 |
|  | Validation | 0.799 | 0.771 | 0.790 | 0.830 | 0.824 - 0.835 |
| Logistic Regression | Training | 0.804 | 0.800 | 0.794 | 0.852 | 0.849 - 0.856 |
|  | Validation | 0.799 | 0.771 | 0.789 | 0.830 | 0.824 - 0.835 |
| Naive Bayes | Training | 0.813 | 0.781 | 0.766 | 0.851 | 0.847 - 0.854 |
|  | Validation | 0.831 | 0.701 | 0.764 | 0.815 | 0.810 - 0.821 |

Abbreviations: AUC, area under the receiver operating characteristic curve; CI, confidence interval; ML, machine learning; LightGBM, Light Gradient Boosting Machine; XGBoost, Extreme Gradient Boosting.

**eFigure 1. Workflow of data processing, trajectory modeling, and ML model development for *H. pylori* infection prediction.**

**
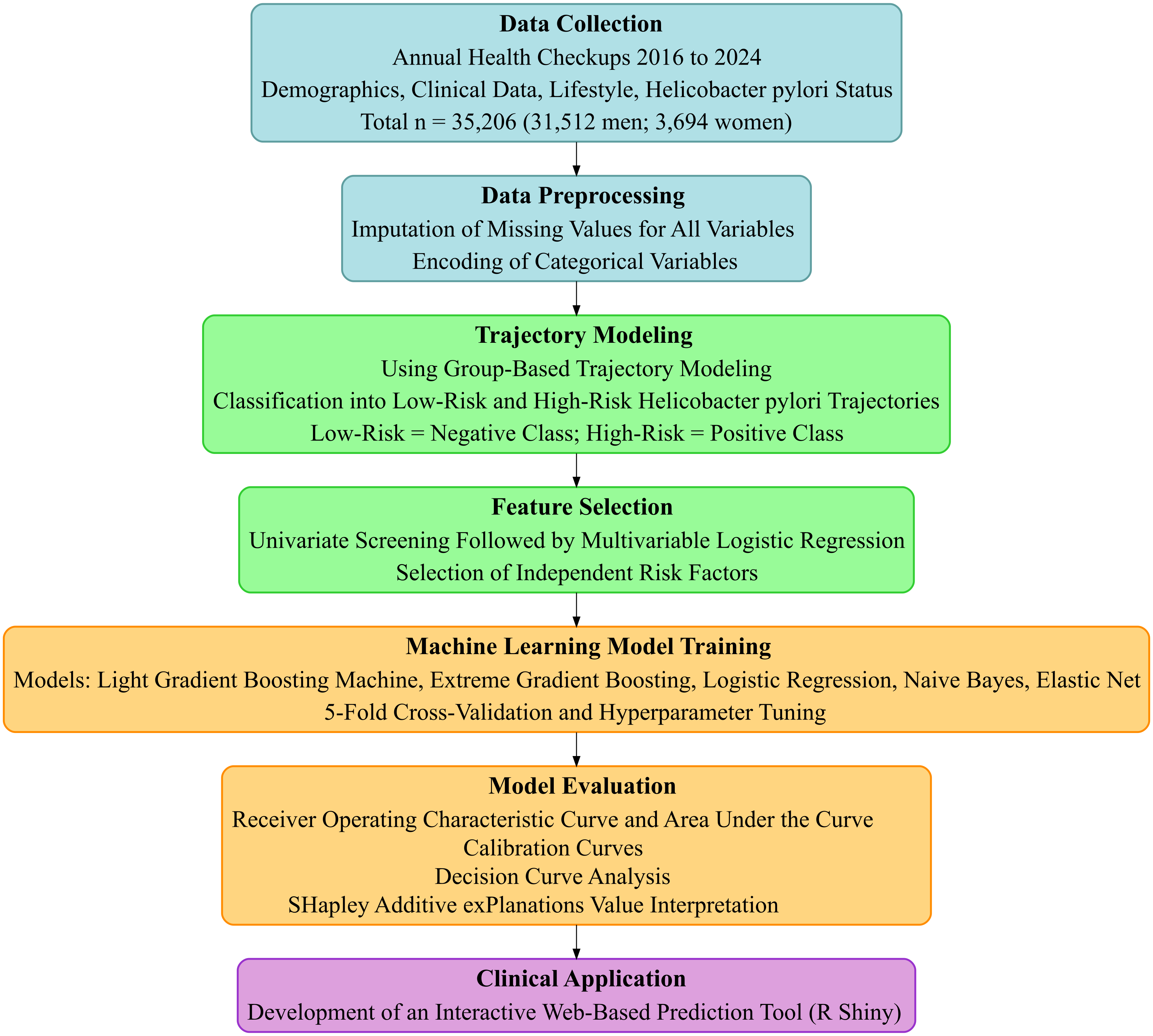
**

**eFigure 2. Subgroup analysis stratified by alcohol consumption.** Forest plot showing interaction effects on *H. pylori* infection risk. OR:odds ratio.

**
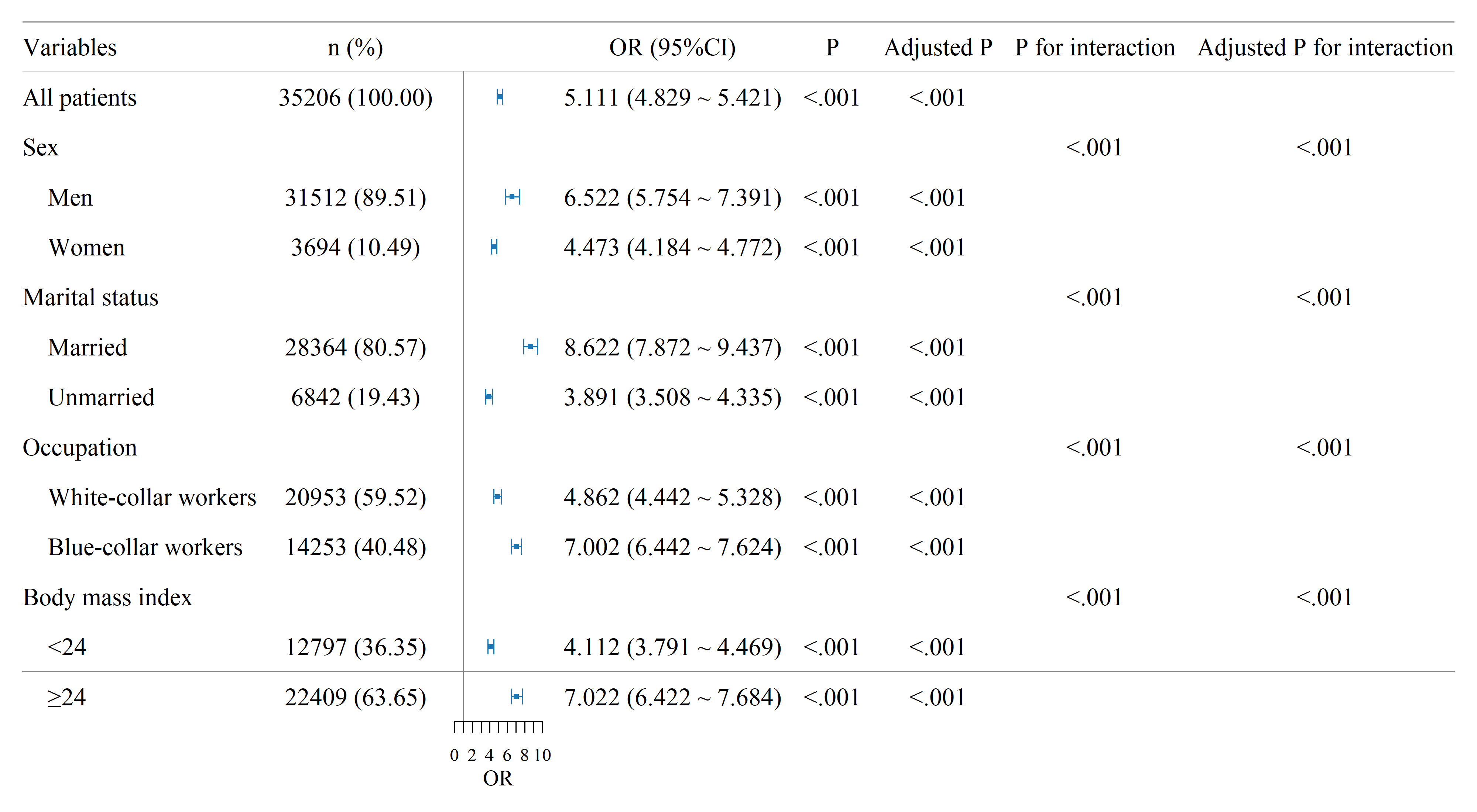
**

**eFigure 3. Model performance in the training cohort.** (A) Baseline trajectory plot; (B) ROC curve; (C) Calibration curve; (D) DCA. AUC: area under the receiver operating characteristic curve; DCA: decision curve analysis; ROC: receiver operating characteristic.

A
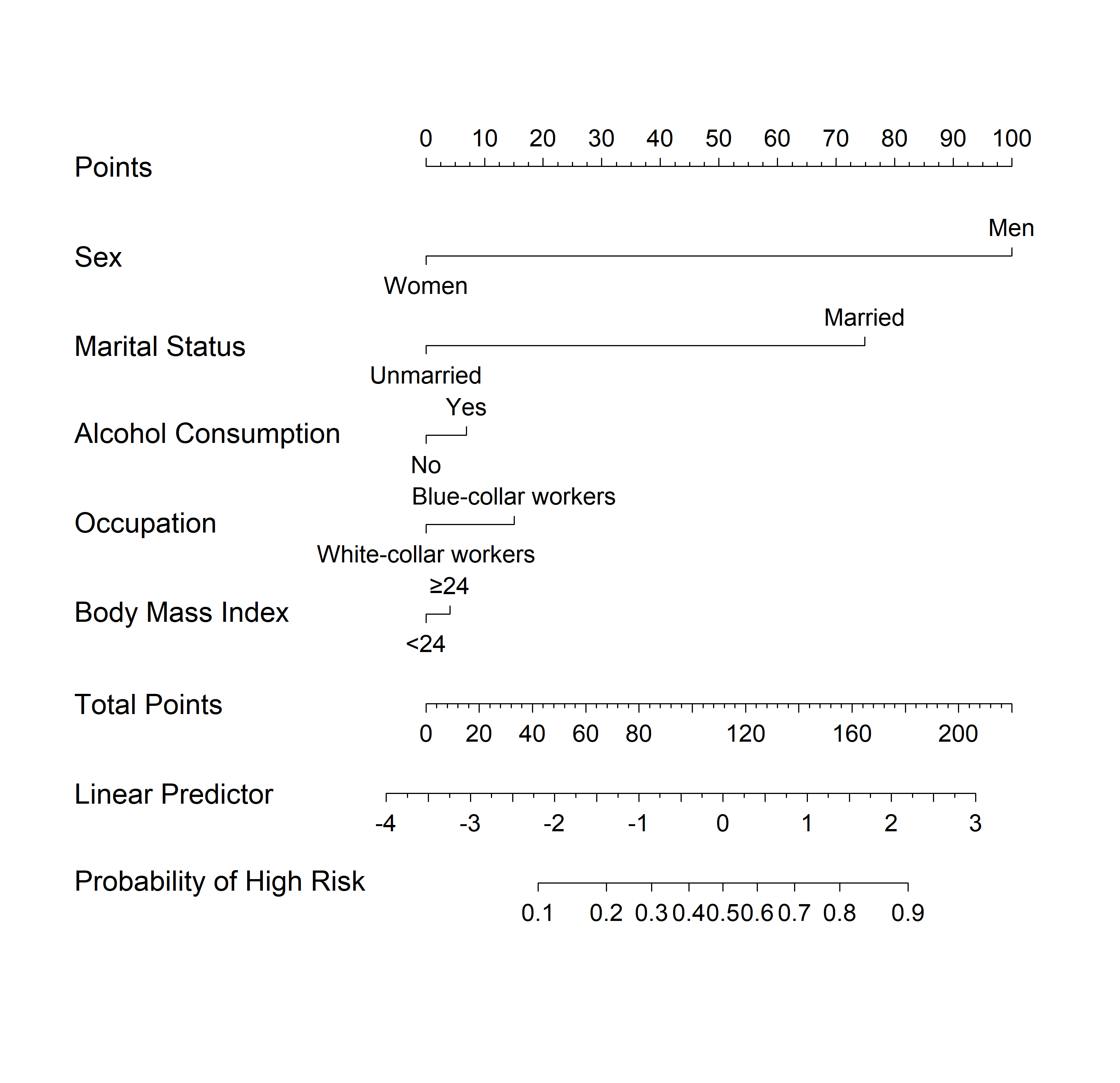
B
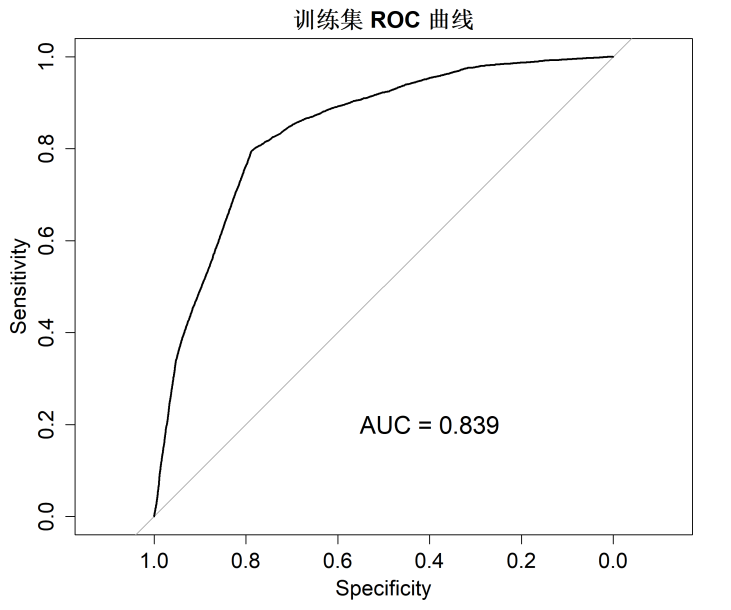


C
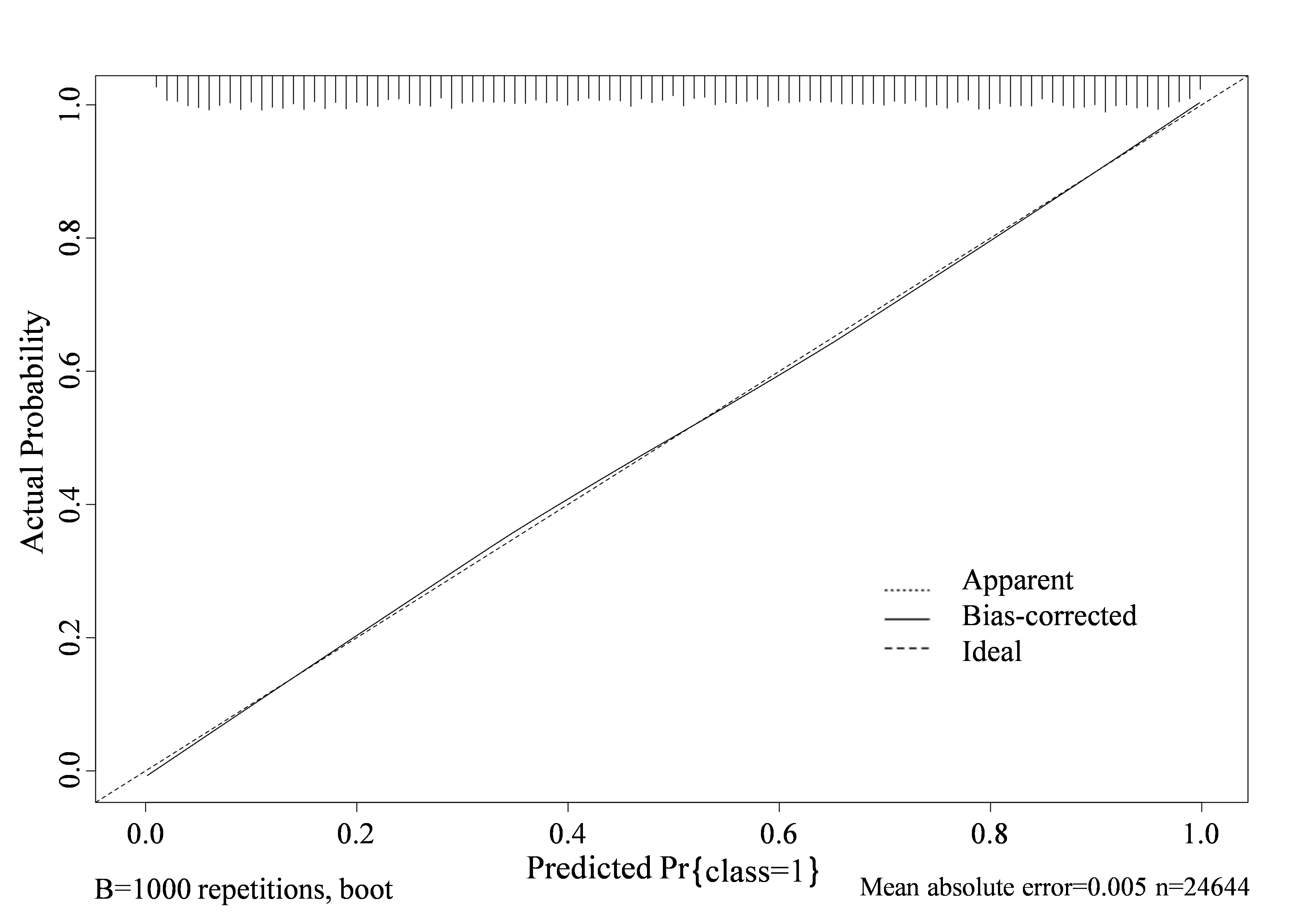
D
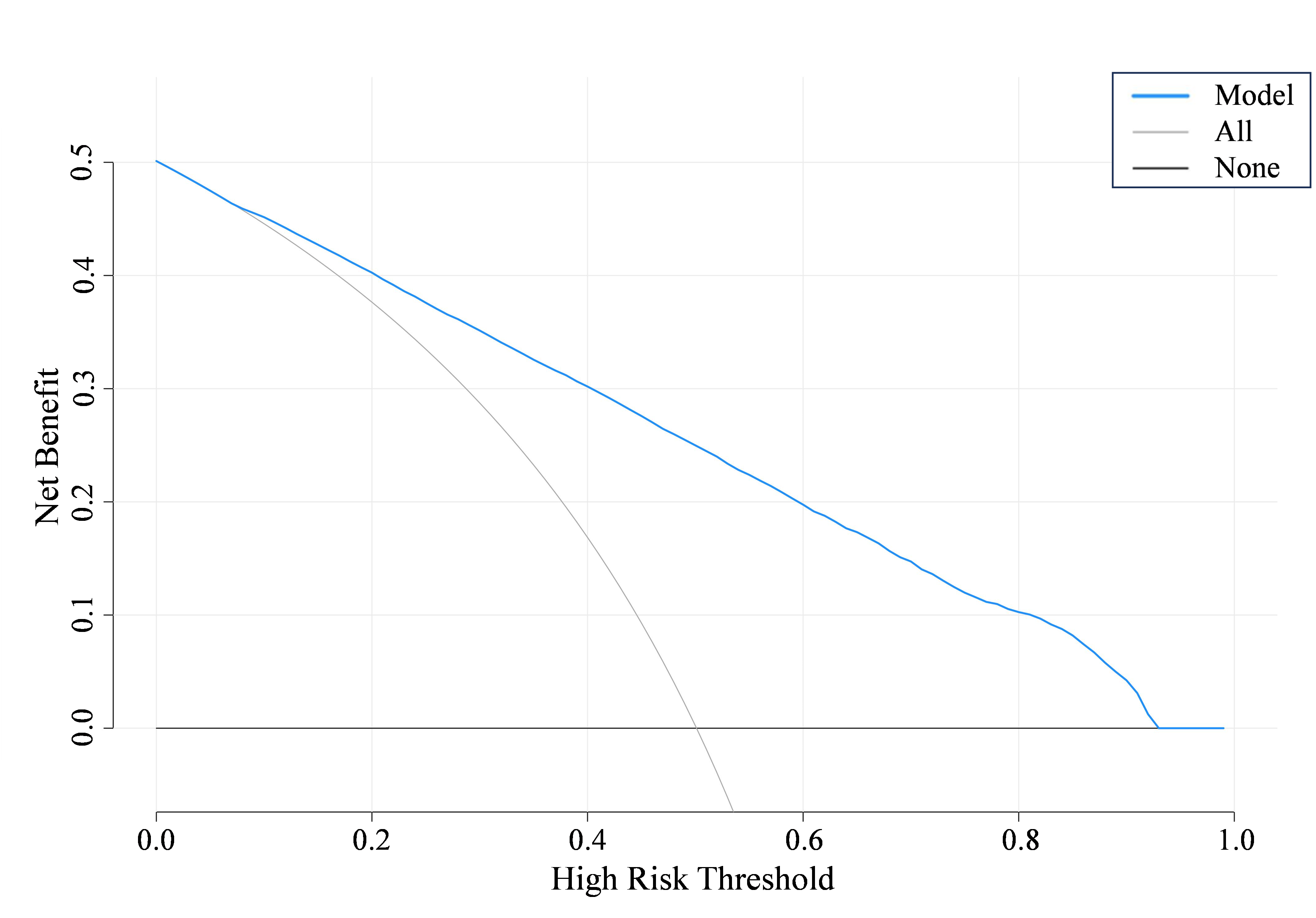


**eFigure 4. Model performance in the validation cohort.** (A) ROC curve; (B) Calibration curve; (C) DCA. AUC: area under the receiver operating characteristic curve; DCA: decision curve analysis; ROC: receiver operating characteristic.

A
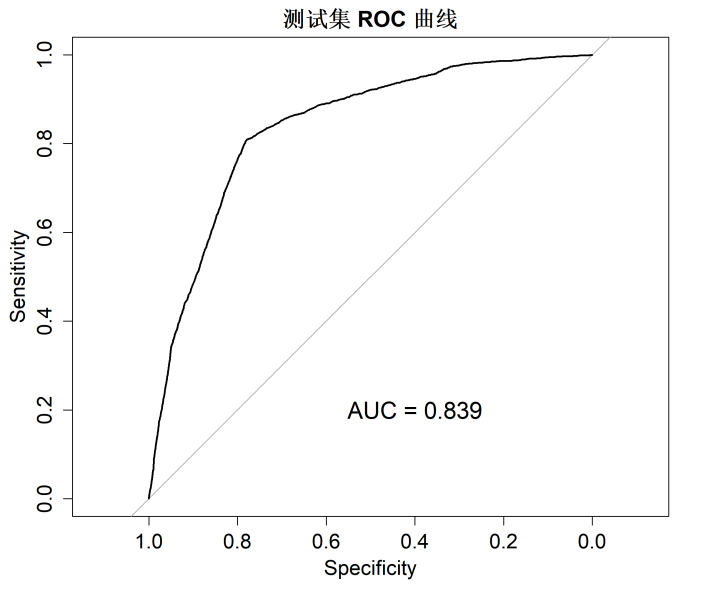
B
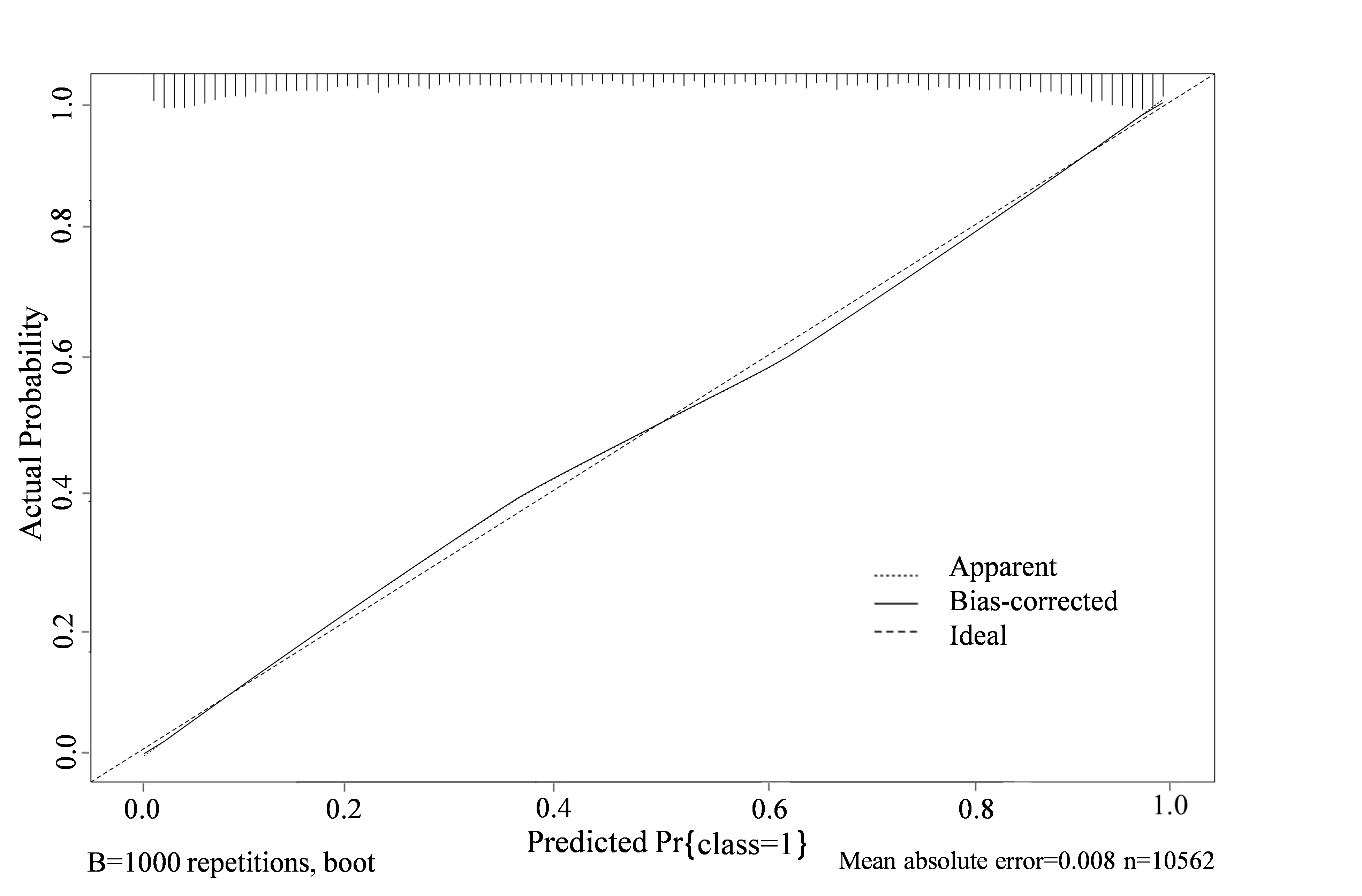


C
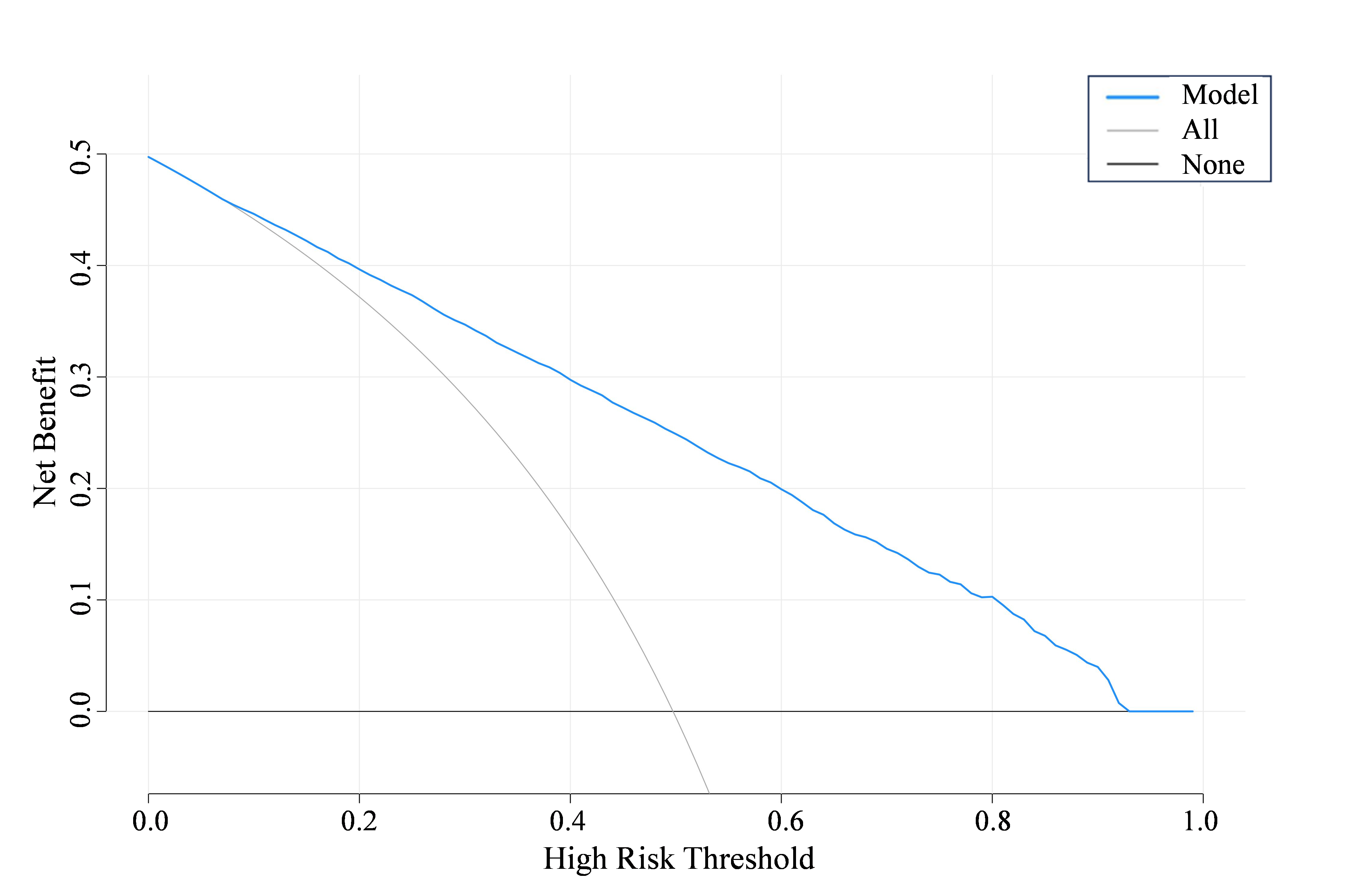


**eFigure 5. Temporal training set evaluation (2016–2020).** (A) Calibration curve; (B) DCA; (C) ROC curves for five ML models. AUC: area under the receiver operating characteristic curve; DCA: decision curve analysis; LightGBM: Light Gradient Boosting Machine; ROC: receiver operating characteristic; XGBoost: Extreme Gradient Boosting; ML: machine learning.

A
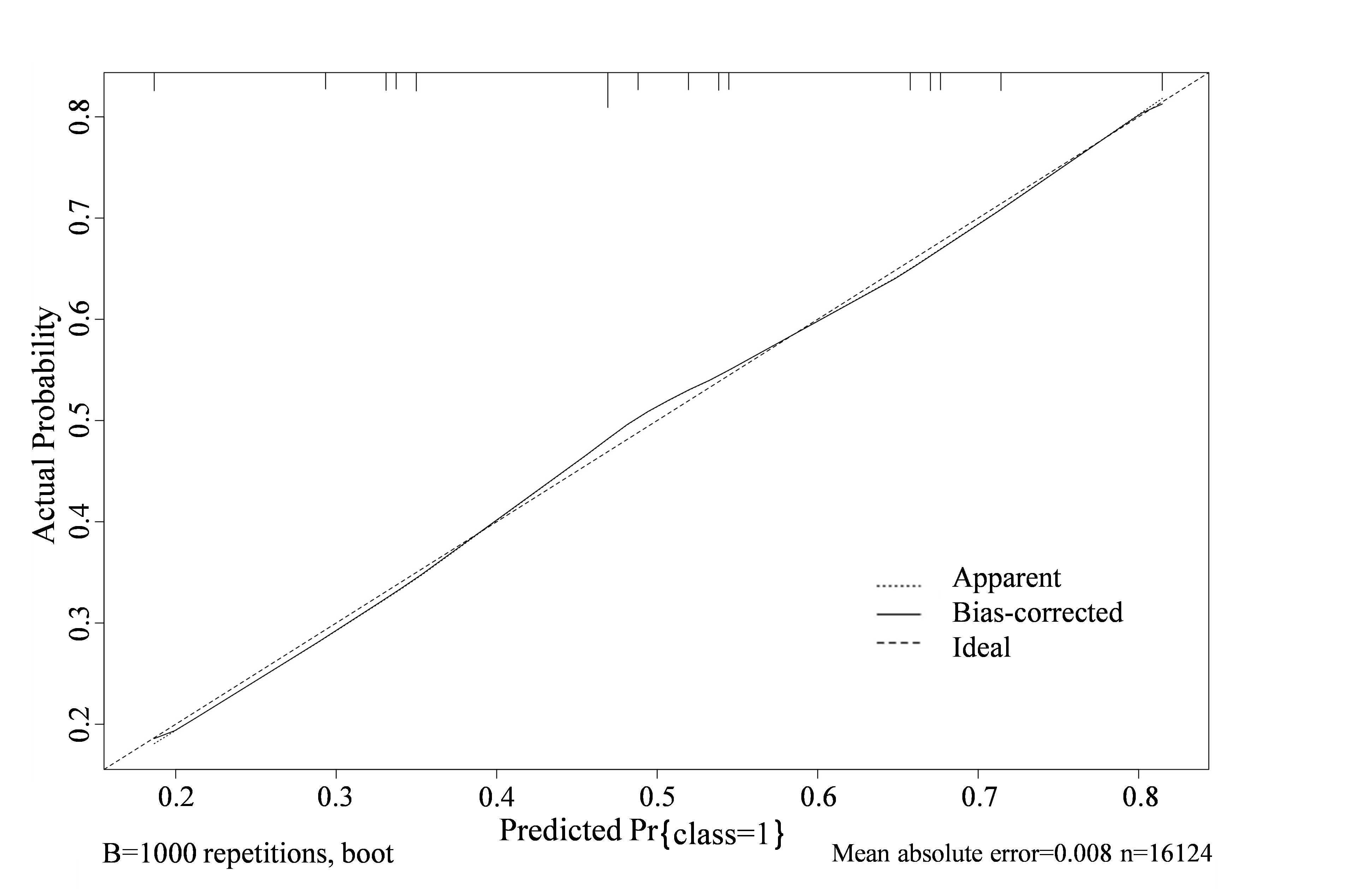
B
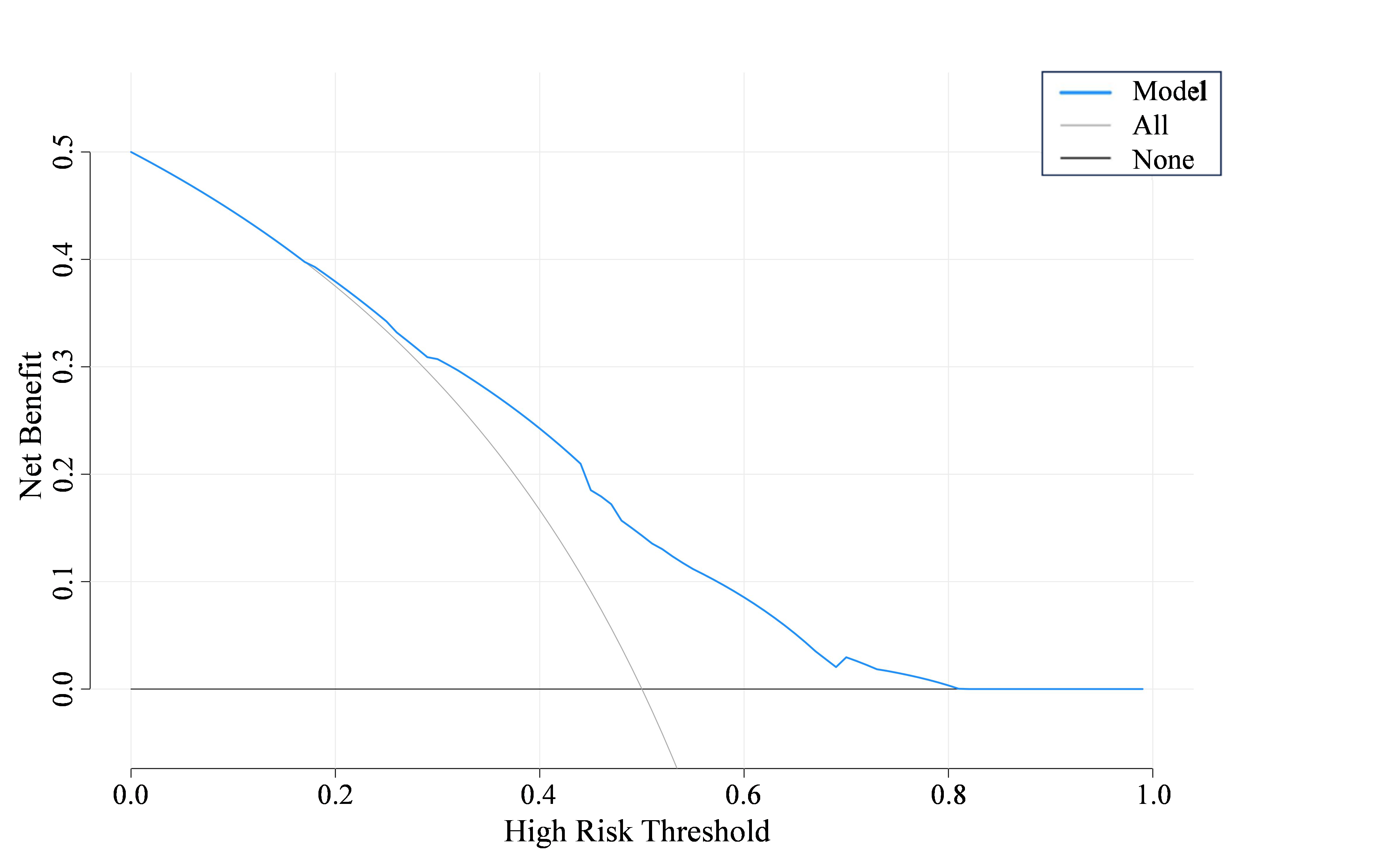


C
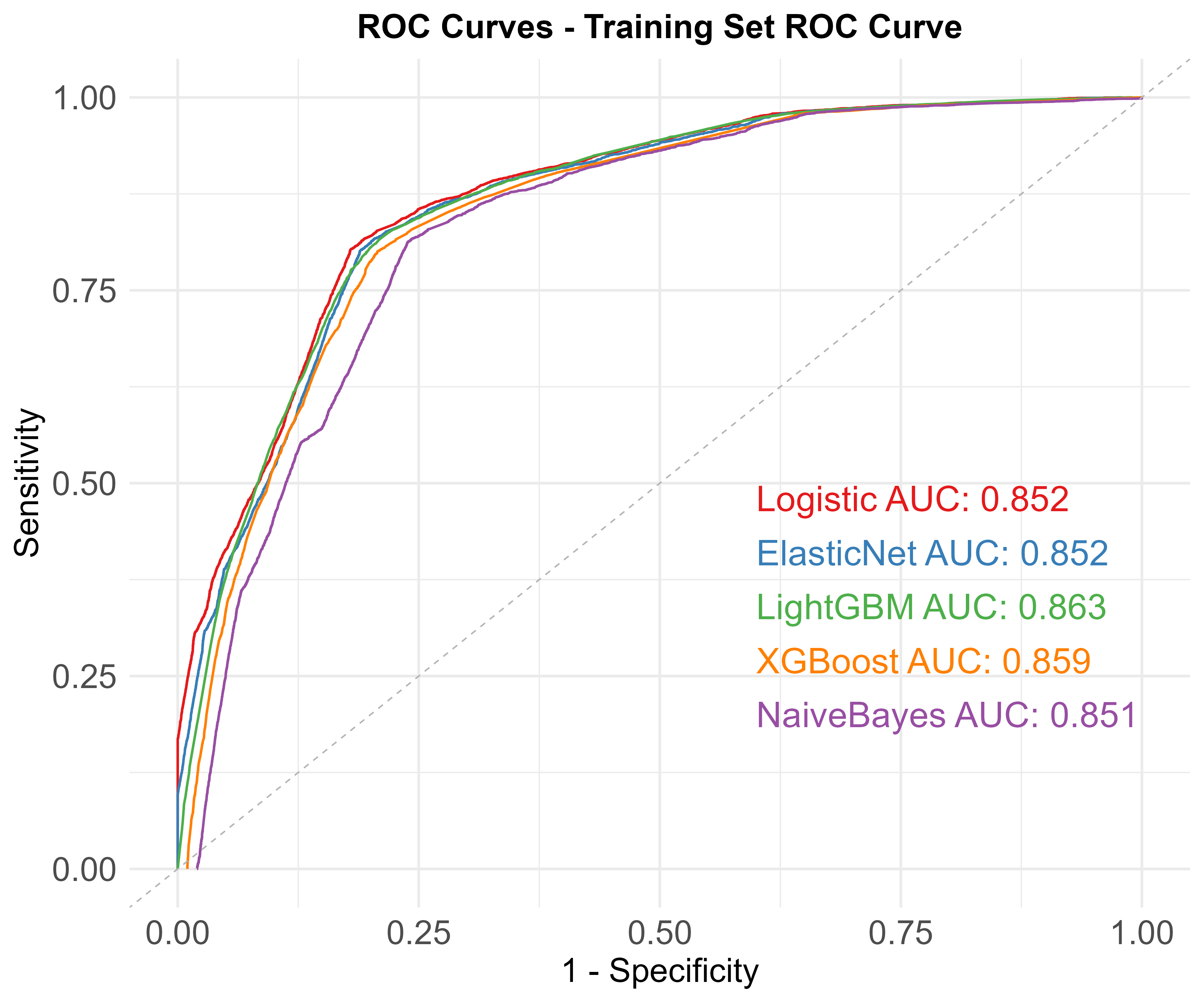


**eFigure 6. Temporal validation set evaluation (2021–2024).** (A) Calibration curve; (B) DCA; (C) ROC curves for five ML models. AUC: area under the receiver operating characteristic curve; DCA: decision curve analysis; LightGBM: Light Gradient Boosting Machine; ROC: receiver operating characteristic; XGBoost: Extreme Gradient Boosting; ML: machine learning.

A
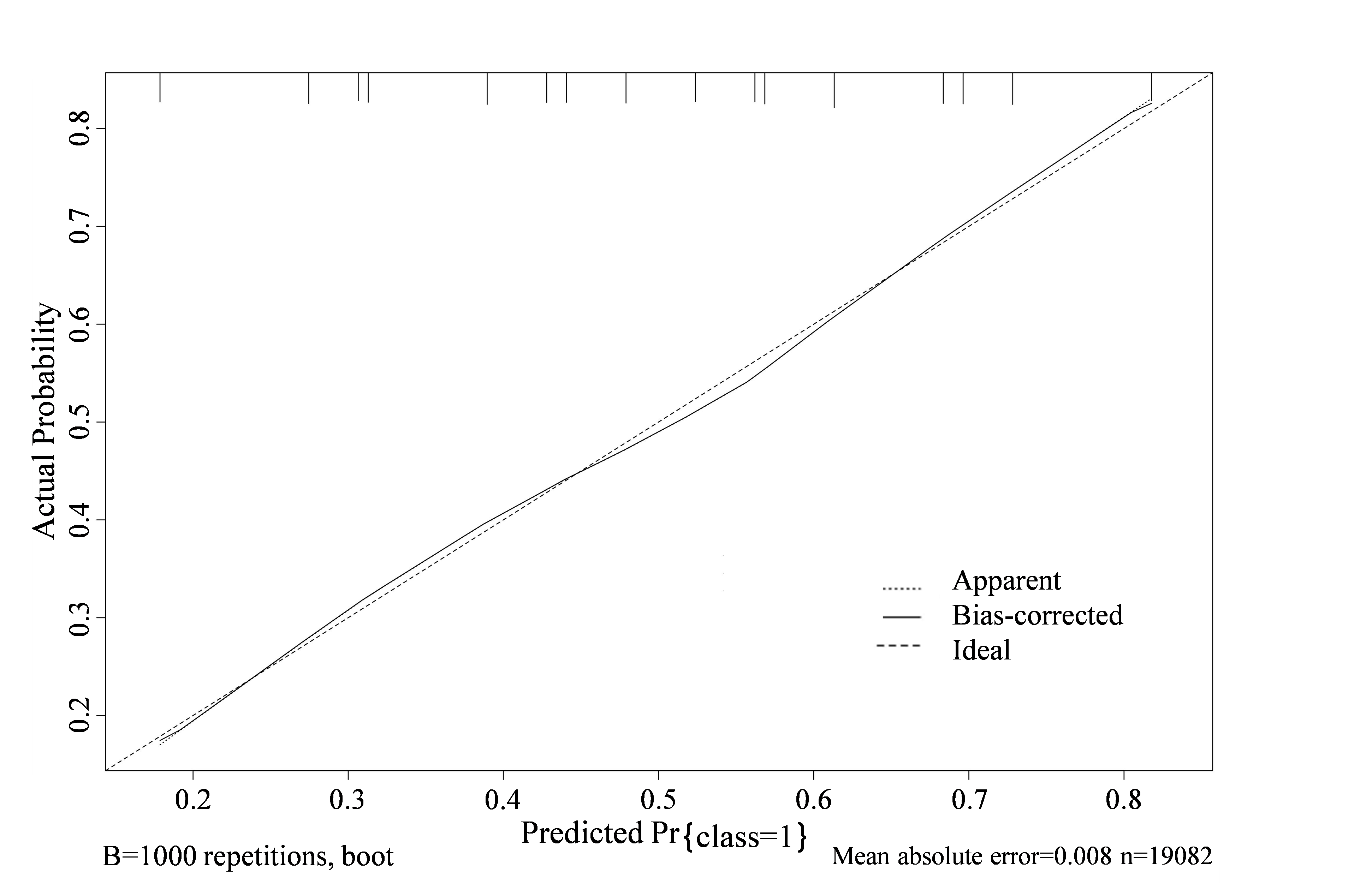
B
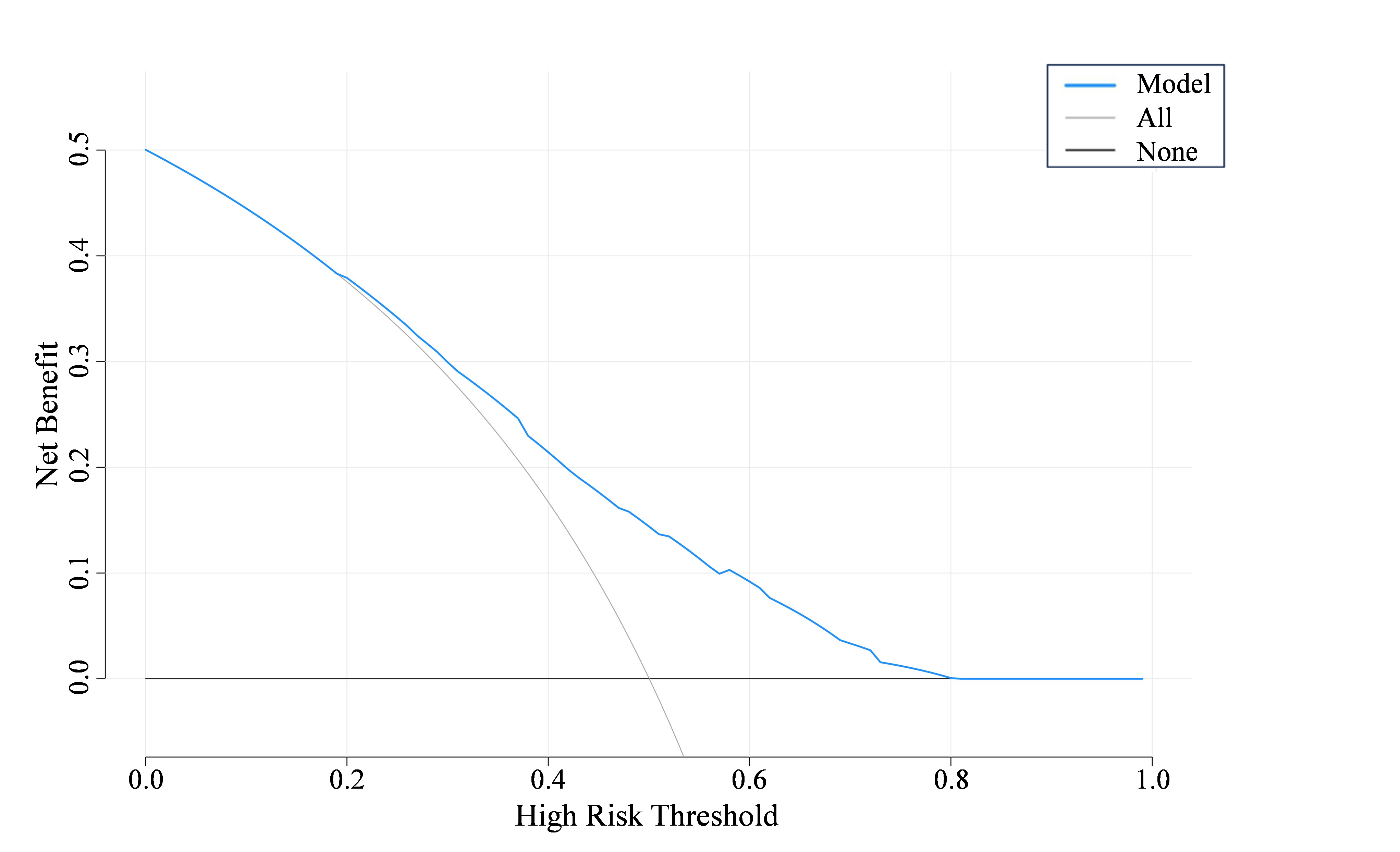


C
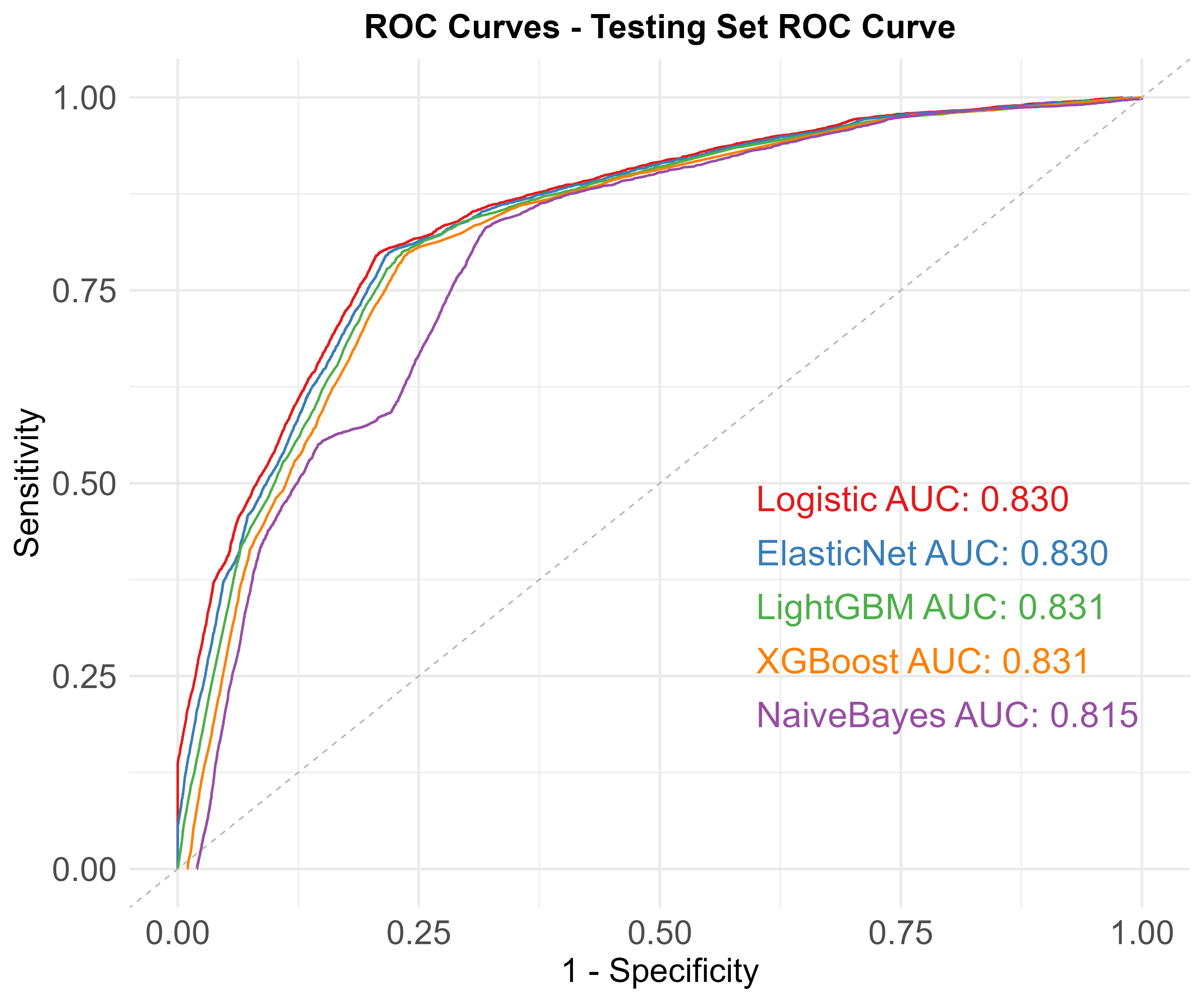


**eFigure 7. Web-based prediction tool interface.** Interface of the R Shiny prediction application with example usage demonstration.


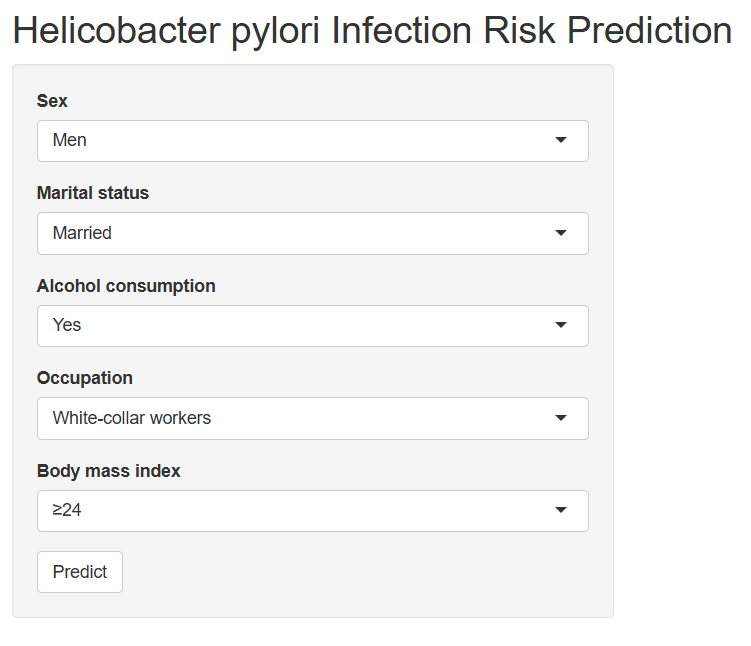

Supplement: Supplementary file 1 [file Data_Sheet_1.docx]
